# The Impact of the Critical Care Resuscitation Unit on Quaternary Care Accessibility for Rural Patients: A Comparative Analysis

**DOI:** 10.1155/2024/9599855

**Published:** 2024-08-22

**Authors:** Quincy K. Tran, Anastasia Ternovskaia, Jessica V. Downing, Minahil Cheema, Taylor Kowansky, Isha Vashee, Jasjot Sayal, Jasmine Wu, Aditi Singh, Daniel J. Haase

**Affiliations:** ^1^ Department of Emergency Medicine University of Maryland School of Medicine, Baltimore, MD, USA; ^2^ The R Adams Cowley Shock Trauma Center University of Maryland School of Medicine, Baltimore, MD, USA; ^3^ The Research Associate Program in Emergency Medicine and Critical Care Department of Emergency Medicine University of Maryland School of Medicine, Baltimore, MD, USA; ^4^ University of Maryland School of Medicine, Baltimore, MD, USA

## Abstract

**Background:**

Previous research suggests that patients from rural areas who are critically ill with complex medical needs or require time-sensitive subspecialty interventions face worse healthcare outcomes and delays in care when compared to those from urban areas. The critical care resuscitation unit (CCRU) at our quaternary care center was established to expedite the transfer of critically ill patients or those who need time-sensitive intervention. This study investigates if disparities exist in treatments and outcomes among patients transferred to the CCRU from rural versus urban hospitals.

**Methods:**

This is a retrospective study of adult, nontrauma patients admitted to the CCRU via interhospital transfer from outside facilities from January 1 to December 31, 2018. Patients transferred from within our institution or with missing clinical data were excluded. Multivariable logistic regressions were performed to measure the association between patients' demographic and clinical factors with in-hospital mortality.

**Results:**

We analyzed 1381 nontrauma patients, and 484 (35%) were from rural areas. Median age was 59 [47–69], and 629 (46%) were female. Median sequential organ failure assessment was 3 ([1–6], *p*=0.062) for both patients transferred from urban and rural hospitals. There was no significant difference between groups with respect to most demographic and clinical factors, as well as types of interventions after CCRU arrival, including emergent surgical interventions within 12 hours of arrival at the CCRU. Rural patients were more likely to be transferred for care by the acute care emergency surgery service than were patients from urban areas and were transferred over a significantly greater distance (difference of 53 kilometers (km), 95% CI: –58.9–51.7 km, *P* < 0.001). Transfer from rural areas was not associated with increased odds of in-hospital mortality (OR: 0.90, 95% CI: 0.60, 1.36; *P*=0.63).

**Conclusion:**

Thirty-five percent of patients transferred to the CCRU came from rural areas, which house 25% of the state population of Maryland. Patients transferred from rural counties to the CCRU faced greater transport distances, but they received the same level of care upon arrival at the CCRU and had the same odds of in-hospital mortality as patients transferred from urban hospitals.

## 1. Introduction

The provision of advanced medical care to critically ill patients poses significant challenges, especially for those residing in rural areas [1]. Disparities in healthcare access between urban and rural regions are well documented and often result in delayed or suboptimal care for rural populations [2]. In the context of this study, rural areas are defined by lower population density and greater distance from healthcare facilities, while urban areas are characterized by higher population density and proximity to comprehensive healthcare resources [3]. This disparity is particularly pronounced in access to specialized critical and quaternary care resources, which are usually concentrated in urban centers [2]. Interhospital transfers over significant distances are frequently required to connect rural patients to these resources, as oftentimes transfer to tertiary care centers leads to reduced mortality [4], and the time needed to coordinate and complete these transfers can have significant implications for patients with critical and time-sensitive conditions.

Maryland is a relatively small state, and thus its rural areas are in much closer proximity to urban resources compared to larger and more rural states in the US. However, rural populations in smaller states, primarily along the East Coast, still face significant barriers to accessing advanced medical care. These populations are often overlooked in discussions about rural healthcare disparities, which tend to focus on larger, more remote rural areas largely located geographically towards the midwest [2]. This study specifically focuses on rural communities in smaller states, highlighting their unique challenges and the importance of addressing healthcare access for this population.

Maryland's diverse geographical landscape presents unique challenges in providing equitable access to advanced medical care. The state's two quaternary care centers are located in Baltimore, creating significant distance barriers for patients in rural areas, particularly on the eastern shore and in the western regions [5]. The eastern shore is separated from Baltimore by the Chesapeake Bay, with the Bay Bridge serving as a critical yet potentially congested route. The distance from Worcester County to Baltimore is approximately 130 miles, a journey that can be prolonged by traffic conditions. Similarly, patients from Garrett County face a distance of around 180 miles to reach these centers. These logistical barriers highlight the need for a well-coordinated system of interhospital transfers and other innovative solutions to bridge the gap in access to specialized medical services [6]. The reliance on Baltimore's quaternary care centers by the state's rural population underscores the importance of addressing these challenges to ensure equitable healthcare access for all Maryland residents.

The critical care resuscitation unit (CCRU) at the University of Maryland Medical Center (UMMC) was designed to improve Marylanders' access to critical and quaternary care by expediting interhospital transfers, coordinating advanced preparation for emergent procedures, and advancing the provision of specialized critical care from the moment a patient enters the hospital or is picked up by the transport team at an outside facility [7]. This study examines whether the CCRU reduces disparities in access to specialized medical care and hospital outcomes for patients with specialized or critical care needs transferred from rural settings throughout the state. By investigating the presence of previously identified rural/urban health disparities within the CCRU's operations, this study aims to determine if the CCRU model can potentially improve accessibility and reduce disparities in healthcare access for Maryland's rural population. The results of this investigation could shape future strategies for managing interhospital transfers and enhancing the quality of care for patients from rural areas, who often face unique challenges in accessing advanced medical services.

## 2. Methods

### 2.1. Study Setting

The CCRU is a 6-bed intensive care unit (ICU)-based resuscitation unit and is located in the R Adams Cowley Shock Trauma Center at the University of Maryland Medical Center (UMMC) in Baltimore, Maryland. This unit is responsible for the triage, initial resuscitation, and coordination of care for patients transferred from other hospitals across the state of Maryland. When a physician from an outside hospital identifies a patient as a potential candidate for advanced interventions (such as advanced mechanical life support or surgical or endovascular therapies) or has critical care needs exceeding the capacity of their current institution, they can consult the CCRU and the relevant specialists through the Maryland Access Center, a 24/7 centralized hub capable of coordinating services across the University of Maryland Medical System. The patient's appropriateness for transfer and an initial care plan is determined based on a joint conversation between the referring physician, CCRU physician, and specialists; transfer priority is determined by the CCRU physician based on the acuity of the patient's needs, available resources, and other incoming transfers. On arrival to the CCRU, the patient is reassessed by the CCRU team, who admits the patient and identifies any needed workup or interventions, and by the accepting specialist. Patients requiring emergent interventions are rapidly prepared for and transported to interventions as indicated. Certain patient populations, such as those requiring evaluation for venoarterial or venovenous extracorporeal membrane oxygenation (VA or VV ECMO), neurosurgical or cardiac surgery interventions, and mechanical thrombectomy for cerebrovascular accident due to large vessel occlusion, are transferred to UMMC exclusively through the CCRU, where others, such as those requiring subspecialty medical intensive care, are transferred through the CCRU only when a bed is not immediately available to accept the patient in their “destination unit” (such as the medical or surgical ICU). From 2013 to 2018, 25% of all transfers to UMMC occurred through the CCRU [8]. Prior research has shown that patients transferred through the CCRU faced lower in-hospital mortality compared to those transferred to a traditional ICU within UMMC. [9]. Other specifics regarding the CCRU physician, advanced practice provider (APP), and nurse staffing have been previously described in detail [10].

### 2.2. Study Design and Patient Selection

This was a retrospective study of all adult nontrauma patients admitted through the CCRU via interhospital transfer between January 01, 2018, and December 31, 2018. Trauma patients at our institution are primarily admitted through a separate trauma resuscitation unit (TRU) at the R Adams Cowley Shock Trauma Center and were thus excluded from this study. All patients admitted to the CCRU from our own institution's emergency department or other inpatient units were excluded. Patients with missing time stamps for relevant events before or after CCRU arrival or who had negative time intervals (indicating inaccurate timestamps) were also excluded. We compared data for patients transferred to UMMC from hospitals located in rural versus urban counties as designated by the state of Maryland [11]. The state of Maryland recognizes 18 of the state's 24 jurisdictions as rural areas; up to 25% of the state's residents live in these 18 jurisdictions. Due to its retrospective observational nature, the study was exempted from formal review by the Institutional Review Board at the University of Maryland, Baltimore (HP-00084554).

The aim of the study is to identify disparities in access to specialized medical care and hospital outcomes among patients transferred to the CCRU from rural versus urban hospitals throughout the state of Maryland. In a prior publication, we demonstrated that the CCRU successfully facilitates the timely transfer and access to care based on patient acuity and time sensitivity [12]. Based on this finding, we hypothesize that patients transferred from rural hospitals would receive comparable treatment and face similar outcomes compared with those from urban hospitals. However, we do expect to see some differences in the types of conditions, services, and interventions that these two groups are transferred for, based on the local availability of services.

### 2.3. Study Outcomes

Our primary outcome was in-hospital all-cause mortality. Our secondary outcome was the number of patients requiring urgent surgical interventions, defined by arrival in the operating room within 12 hours of CCRU arrival. Other outcomes included hospital length of stay, discharge disposition, and time intervals from initial transfer request to CCRU bed assignments and from transfer request to CCRU arrival.

### 2.4. Data Collection

Members of the research team were blinded to the study's hypothesis. They were trained to collect data from the hospital's electronic medical record (EMR) by the principal investigator. As part of training, they collected data from patients' charts in sets of 5 until accuracy reached 90% in comparison to data collected by a senior investigator. Up to 5% of data was subsequently randomly checked by a senior investigator for accuracy. Any discrepancies were discussed among team members and corrected by the senior investigators. Data were entered into a standardized Microsoft Excel spreadsheet (Microsoft Corp, Washington, USA).

We collected data regarding patients' demographics (such as age, gender, and past medical history), clinical information (serum lactate levels, hemoglobin, and individual components of the sequential organ failure assessment [SOFA] score) on CCRU arrival, and outcomes (hospital mortality, operations and other interventions during hospitalization, hospital length of stay, and discharge disposition).

Since most laboratory evaluations were part of the standard of clinical care for CCRU patients, we anticipated minimal missing data.

### 2.5. Statistical Analysis

We did not perform a formal sample size calculation; we anticipated enrollment of approximately 1500 patients for the full calendar year, with an estimated 25% from rural areas (in line with the state's population distribution). We expected this would provide a sufficient sample size to compare urban and rural patients.

Patients' demographic and clinical information were presented using descriptive analyses. Prior to analyses, histograms of continuous independent variables were inspected to determine their patterns of distribution. Continuous independent variables were presented with mean (±standard deviation (SD)) or median (interquartile range (IQR)) according to their distributions and were compared by the *t*-test or Mann–Whitney *U* test. Categorical variables were expressed as *N* and percentage and were compared with chi-square tests. Comparisons of independent variables between groups (urban vs. rural) were also expressed with differences and their associated 95% confidence intervals (95% CIs).

We conducted multivariable logistic regressions to determine the association between nontrauma patients' demographic (including initial presentation to rural hospitals) and clinical factors and in-hospital mortality. We selected independent variables (Appendix A) *a priori* as those identified by previous literature as predictors of in-hospital mortality [13]. Results from multivariable logistic regressions were expressed as odds ratio (OR), 95% CI, and *p* value. Multicollinearity was assessed using the variance inflation factor (VIF). Factors with VIF >5 were considered to have a high collinearity and were eliminated from the models. The goodness of fit of the models was assessed with the Hosmer–Lemeshow analysis, of which *p* value >0.05 indicated good fit of the data. The performance of the models was evaluated with the area under the receiver operating curve (AUROC). A model with AUROC approaching 1.0 would indicate excellent discriminatory capability between dichotomous outcomes (survivor vs. nonsurvivor).

Sensitivity analysis was performed using a multivariable ordinal logistic regression with the outcome of patients' disposition at hospital discharge. Discharge dispositions were ranked in the order of 0 (discharge home directly), 1 (any rehabilitation center), 2 (skilled nursing home/facility), and 3 (hospice/death). Results from the ordinal regression were expressed as OR, 95% CI, and correlation coefficient (corr. coeff). A positive correlation indicates an increased likelihood of the lowest rank outcome (0, discharge home), while a negative correlation coefficient indicates an increased likelihood of the highest rank outcome (3, dead/hospice) more likely.

The majority of missing data pertained to the following laboratory markers: two bilirubin measurements, two white blood cell counts, one hemoglobin measurement, and 220 troponin measurements. Most of these markers are routine for all patients in the CCRU, but troponin is ordered primarily for patients with cardiovascular symptoms. Our analysis centered on patient conditions assessed through the SOFA score; since troponin measurements are not included in the SOFA scoring system, their absence did not impact the score. Of the laboratory markers contributing to the SOFA score (bilirubin, white blood cell count, and hemoglobin), only five values were missing. These missing values were imputed as “normal.”

All descriptive analyses and multivariable logistic regressions were performed with Minitab version 20 (https://www.minitab.com, State College, Pennsylvania, USA). All statistical analyses with *p* value <0.05, except the Hosmer–Lemeshow test as discussed above, were considered statistically significant.

## 3. Results

### 3.1. Demographics

**Figure 1:**
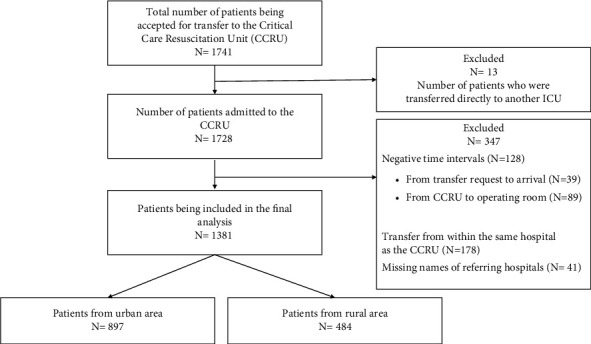
Patient selection diagram.

**Figure 2:**
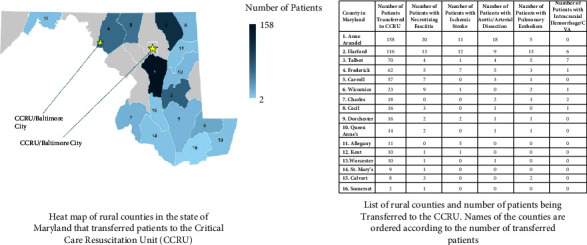
Heat map of rural counties transferring patients to the critical care resuscitation unit (CCRU). The numbers on the map correspond to the county identity in the table. Heat map was generated from our own dataset for this study using Microsoft Excel.

There were a total of 1731 transfer requests during our study period; we included 1381 nontrauma patients in the final analysis (Figure 1) who were transferred from other hospitals. Eight hundred and ninety-seven (65%) patients were transferred from urban areas, while 484 (35%) were from rural areas (Table 1). The median age of the population was 59 (47–69) years, and 629 (46%) patients were female. Most of the patients' demographic and clinical factors did not vary significantly between groups (Table 1). However, patients from rural areas were transported over a significantly longer ground distance during interhospital transfer to the CCRU (difference of 53 kilometers, 95% CI: 51.7–58.9 km, *p*  <  0.001) and faced longer transport times (difference of 33 minutes, 95% CI: 22–45 min, *p* < 0.001). A higher percentage of patients from rural hospitals were transferred for evaluation and treatment by the acute care emergency surgery service when compared to those from urban hospitals (16% *v*s 8%, difference of 8%, 95% CI: 4%–12%, *p* < 0.001) (Figure 2).

While higher percentages of patients transferred from urban areas were treated with continuous infusions prior to arrival at the CCRU (difference of 8%, 95% CI: 3–13%, *p*=0.002), there were no significant differences in interventions after CCRU arrival (Table 2(a, b)). The percentage of patients undergoing emergent surgical interventions within 12 hours of arrival at the CCRU was similar between groups: 23% (205) of urban patients and 25% (123) of rural patients (*p*=0.29, Table 2(a, b)). In-hospital mortality rates and hospital length of stay were also similar between groups (Table 2(a, b)).

### 3.2. In-Hospital Mortality

The prevalence of in-hospital mortality was 14% among rural patients, compared to 16% among patients transferred from urban areas (*p*=0.43), and patients transferred from rural areas did not face higher odds of in-hospital mortality (OR: 0.90, 95% CI: 0.60–1.36, *p*=0.63; Table 2(a, b)). However, longer transport distance from the sending facility of transport to the CCRU was also associated with higher odds of unfavorable discharge disposition, including death or discharge to hospice (corr. coeff: −0.01, OR: 1.00, 95% CI: 0.99–1.00, *p* < 0.001; Appendix C).

Higher SOFA score (corr. coeff: −0.16, OR: 0.86, 95% CI: 0.82–0.89, *p* < 0.001) was associated with higher odds of unfavorable discharge disposition, including death or discharge to hospice, as was the need for emergent surgery (corr. coeff: −0.31, OR: 0.73, 95% CI: 0.55–0.95, *p*=0.02), mechanical ventilation initiated prior to CCRU arrival (corr. coeff: −0.34, OR: 0.71, 95% CI: 0.53–0.96, *p*=0.03), higher age (corr. coeff: −0.03, OR: 0.97, 95% CI: 0.96–0.97, *p* < 0.001), and higher serum lactate (corr. coeff: −0.12, OR: 0.89, 95% CI: 0.84–0.94, *p* < 0.001; Appendix C). Multivariable logistic regressions demonstrated that each increment in SOFA score at CCRU arrival was associated with 21% increased odds of in-hospital mortality (OR: 1.21, 95% CI: 1.15–1.28, *p* < 0.001; Appendix C). SOFA score was not significantly different between patients transferred from urban vs rural areas (Table 1).

## 4. Discussion

This single-center, retrospective study investigated the outcomes of nontrauma patients who were transferred from rural areas within the state of Maryland to a specialized ICU-based resuscitation unit in downtown Baltimore, MD, and compared them with those of patients transferred from urban parts of the state. Our study found no significant difference between the two groups with respect to in-hospital mortality or hospital length of stay. The CCRU was designed to expand and expedite access to critical and quaternary care statewide. Prior studies from our group have demonstrated that the unit has increased the number of patients transferred to our institution while decreasing overall times from transfer request to arrival at UMMC, and for patients requiring urgent surgical intervention, to arrival in the operating room, and has decreased mortality for patients transferred through the CCRU when compared to those transferred to a traditional ICU [7, 14, 15]. We cautiously interpret the findings of this study to suggest that the CCRU may also function to reduce disparities in access to quaternary and specialized critical care faced by residents of rural counties in our state.

Given the highly and increasingly specialized and resource-intensive nature of critical care and subspecialty surgical care, it is not unexpected to see a large urban-rural divide, nor to expect that this divide will continue to deepen. This is likely to be compounded by increasing emergency department boarding of critically ill patients, which has been characterized as stretching small critical access hospitals well past their capacity to provide comprehensive and high-quality care [1]. Prior research has demonstrated that patients presenting to rural hospitals for trauma [8] and a variety of medical conditions [9, 10, 16, 17] are more likely to require interhospital transfer and to face worse outcomes than their urban counterparts. Our findings further support these claims. While approximately 25% of Maryland's population resides in rural regions, 35% of patients transferred to the CCRU were transferred from hospitals in rural counties. Patients were most often transferred for specialized care and interventions only available at quaternary centers, such as neurosurgery (intracranial hemorrhage), cardiac surgery (acute aortic disease), or neurology interventional radiology (ischemic stroke requiring thrombectomy; Appendix C). A significantly higher proportion of patients from rural areas were transferred to be cared for by the acute care emergency surgery service; this directly highlights a likely disparity in access to emergency general surgical care between our urban and rural counties. This disparity has been suggested by prior studies as well [18, 19].

Rapid and coordinated transfer of rural patients to quaternary and subspecialty centers is a key component to addressing these disparities. Prior studies have shown that, primarily due to longer transport distances and times, patients from rural areas often face worse outcomes than those from urban areas, even when cared for at the same facilities. A study of trauma patients in Western Australia found that patients from rural areas waited an average of 11.6 hours from the time of injury to definitive care, compared with approximately 1 hour for patients in urban areas [8]. Within the U.S., it has been shown that the time from EMS activation to hospital arrival for patients with STEMI was significantly higher among patients in rural areas, even after accounting for total mileage [20]. Our study found that patients transferred from rural areas faced longer transport distances, and our sensitivity analysis demonstrated that longer transport distance was associated with higher odds of in-hospital mortality. This finding is in line with those of the studies discussed above, which examined the impact of time and distance from the patient in the field to definitive hospital care. However, our study did not identify a disparity in outcomes between patients transferred from rural or urban areas. This may reflect a lack of adequate power in our study, or the impact of utilization of air transportation and/or coordination with transport medical providers.

To our knowledge, this is the first study directly comparing the outcomes of patients from urban and rural areas undergoing interhospital transfer for definitive care. Prior studies have investigated care provided at and enroute to rural and urban hospitals, the need for IHT among patients at rural and urban hospitals [16, 21], or the outcomes more globally of patients from rural and urban areas at the same tertiary (often urban) medical centers [22]. The comparison of outcomes of patients from rural areas who arrived at a tertiary center via IHT with those of patients from urban areas arriving via direct admission introduces a high risk of bias, as patients arriving via IHT have already been selected as those requiring high care intensity or subspecialty care or intervention, and are thus likely to be more critically ill (and potentially “behind” on their need for intervention) than those directly admitted [23]. This study provides an important first foray into a more apples-to-apples comparison of quaternary care and outcomes for patients from rural and urban areas within a single state and suggests that a specialized critical care resuscitation unit may play a role in optimizing that care.

### 4.1. Limitations

Our study setting and patient population were unique, such that our findings may not be directly generalizable. While patient transportation within the state of Maryland is subject to limitations stemming from the state's geography and its inclusion of the Chesapeake Bay, which limits access to a large section of the state, it is a relatively small state, and many of these challenges can be (at least partially) alleviated by judicious use of air transportation, weather permitting. Our findings may not be applicable to areas of the country in which transport distances for rural patients are significantly longer than those within our state. Furthermore, our institution is one of two major referral centers within our area, and we are unable to determine how referral and transfer patterns to the other center may differ in comparison to those described here. The data for our study were from 2018, and in the last year, we had full access to patients' clinical information prior to the COVID-19 pandemic. At that time, the nursing staff for the CCRU was at an optimal level and enabled a higher volume of transfers. As has been the case across the country, staffing levels declined within the CCRU and throughout our institution during the COVID-19 pandemic and in the period immediately following, which may have impacted patient transfers and outcomes. During the COVID-19 pandemic and extending until 2023, the CCRU faced staffing shortages nationwide. As a result of the high acuity, mixed pathology, and unique process of admitting within the CCRU, new patient intakes are and remain restricted to CCRU-trained nurses exclusively. This policy meant traveling nurses and other ICU personnel were unable to perform this critical function. Consequently, the number of yearly CCRU admissions fell significantly when compared to 2018. In addition, due to the inability to manage multiple patient intakes simultaneously, the time from request to arrival was prolonged. However, staffing at our institution and within the CCRU specifically has improved since late 2023, nearing prepandemic levels.

Finally, our investigation of patient outcomes ended with hospital discharge, and thus did not account for potential disparities in access to postacute care, including rehabilitation and primary and subspecialty outpatient care. Each of these components of care plays a role in long-term patient recovery and outcomes, and access to these services has been previously highlighted as important contributors of health disparities between urban and rural Americans [13, 24, 25].

## 5. Conclusion

Patients who were transferred from a rural county within the state of Maryland to the critical care resuscitation unit at the University of Maryland Medical Center did not have significantly different hospital outcomes than those transferred from urban counties, despite facing longer transport distances and times. Patients transferred from rural areas comprised a higher proportion of transfers relative to the overall proportion of rural residents of the state, highlighting potential disparities in local access. Further research is needed to confirm our observations.

## Figures and Tables

**Table 1:** Demographics of the full population of patients transported from rural or urban areas to the CCRU.

Variables	All patients	Urban transfer	Rural transfer	Difference between group	95% CI	*P* value
	*N*=1381	*N*=897 (65%)	*N*=484 (35%)			
Age	59 [47–69]	58 [46–69]	60 [48–70]	−1	−3.0	0.12

*Gender, N (%)*						
Male	752 (54)	486 (54)	266 (55)	−0.01	−0.06, 0.05	0.78
Female	629 (46)	411 (46)	218 (45)	0.01	−0.05, 0.06	0.78

*Past medical history, N (%)*						
Hypertension	633 (46)	421 (47)	212 (44)	0.03	−0.02, 0.09	0.26
Diabetes	341 (25)	223 (25)	118 (24)	0.005	−0.04, 0.05	0.84
Liver disease	97 (7)	70 (8)	27 (6)	0.02	−0.01, 0.05	0.11
Kidney disease	230 (17)	161 (18)	69 (14)	0.04	−0.01, 0.08	0.07
Heart disease	309 (22)	189 (21)	120 (25)	−0.04	−0.08, 0.01	0.12

*Teaching status of sending facilities, N (%)*						
Teaching facility	443 (32)	429 (48)	14 (3)	0.45	0.41, 0.49	**<0.001**
Nonteaching facility	938 (68)	468 (52)	470 (97)	−0.45	−0.49, −0.41	**<0.001**
Ground distance to the CCRU	44.1 [13.4-75.1]	21.5 [6.1-44.1]	74.8 [53.7-114]	−53.3	−58.9, −51.7	**<0.001**

*Day and time of CCRU arrival, N (%)*						
Daytime (07 : 00–19 : 00)	662 (48)	441 (49)	221 (46)	0.04	−0.02, 0.09	0.21
Nighttime (19 : 01–06 : 59)	719 (52)	456 (51)	263 (54)	−0.04	−0.09, 0.02	0.21
Weekday (Mon-Fri)	1016 (74)	668 (74)	348 (72)	0.03	−0.02, 0.07	0.31
Weekend (Sat-Sun)	365 (26)	229 (26)	136 (28)	−0.03	−0.07, 0.02	0.31

*Laboratory values and clinical indices on CCRU arrival, median (IQR)*						
SOFA score	3 [1–6]	3 [1–6]	3 [1–6]	0	0.0	0.62
Shock index	0.68 [0.54–0.85]	0.67 [0.54–0.84]	0.69 [0.55–0.86]	−0.01	−0.04, 0.01	0.42
Lactate (mmol/L)	1.6 [1.2–2.4]	1.6 [1.2–2.4]	1.6 [1.2–2.3]	0	−0.1.0.1	0.84
Troponin (*μ*g/uL)	0.02 [001−0.08]	0.02 [0.01–0.07]	0.02 [0.01–0.09]	0	0.0	0.12
WBC (×1000 counts/*μ*L)	12.2 [8.8–17]	12 [8.8–17.1]	12.4 [8.9–16.7]	−0.1	−0.8, 0.6	0.77
Hemoglobin (g/dL)	11.4 [9.4–13.3]	11.4 [9.2–13.1]	11.8 [9.8–13.7]	−0.5	−0.8, −0.2	**0.001**

*Accepting specialty atUMMC* ^1^ *, N (%)*						
Cardiac surgery	288 (21)	182 (20)	106 (22)	−0.02	−0.06, 0.03	0.49
Soft tissue surgery	238 (17)	162 (18)	76 (16)	0.02	−0.02, 0.06	0.26
Neurosurgery	188 (14)	133 (15)	55 (11)	0.03	−0.002, 0.07	0.06
Acute care emergency surgery	145 (10)	69 (8)	76 (16)	−0.08	−0.12, −0.04	**<0.001**
Vascular surgery	130 (9)	78 (9)	52 (11)	−0.02	−0.05, 0.01	0.23
Time from transfer request to bed assignment (minutes), median (IQR)	9 [0–74]	9 [0–81]	8 [0–67]	0	0.1	0.28
Time from transfer request to CCRU arrival (minutes), median (IQR)	173 [116–291]	159 [106–282]	196 [140–308]	−33	−45, −22	**<0.001**

Bolded values indicate statistical significance. ^1^Only the top 5 accepting services were listed here. UMMC, University of Maryland Medical Center CCRU, critical care resuscitation unit; CI, confidence interval; SOFA score, the sequential organ failure assessment score; Mon, Monday; Fri, Friday; Sat, Saturday; Sun, Sunday; mmol/L, millimole per liter; ug/uL, microgram per microliter; WBC, white blood cell; g/dL, gram per deciliter; IQR, interquartile range.

**Table 2:** (a) Clinical interventions and outcomes of the full population of patients who were transported from rural and urban areas to the CCRU. (b) Multivariable logistic regression assessing the association between patients' demographic and clinical factors and hospital disposition of dead/hospice.

(a)						
Interventions	All patients	Urban transfer (0)	Rural transfer (1)	Difference between groups	95% CI	*P* value
	*N*=1381	*N*=897	*N*=484			

*Interventions prior to CCRU arrival, N (%)*						
Mechanical ventilation	455 (33)	299 (33)	156 (32)	0.01	−0.04, 0.06	0.68
Any blood transfusion	132 (10)	89 (10)	43 (9)	0.01	−0.02, 0.04	0.53
Any vasopressors	258 (19)	171 (19)	87 (18)	0.01	−0.03, 0.05	0.62
Any infusion	461 (33)	325 (36)	136 (28)	0.08	0.03, 0.13	**0.002**
Having 2 infusions or more	195 (14)	135 (15)	60 (12)	0.03	−0.01, 0.06	0.17

*Type of infusion*						
Insulin	184 (13)	126 (14)	58 (12)	0.02	−0.02, 0.06	0.27
Clevidipine	76 (5)	56 (6)	20 (4)	0.02	−0.01, 0.04	0.08
Nicardipine	104 (8)	85 (9)	19 (4)	0.06	0.03, 0.08	**<0.001**
Esmolol	55 (4)	35 (4)	20 (4)	−0.002	−0.02, 0.02	0.84

*Interventions after CCRU arrival, N (%)*						
Mechanical ventilation	548 (40)	350 (39)	198 (41)	−0.02	−0.07, 0.04	0.49
Any blood transfusion	87 (1411)	57 (6)	30 (6)	0.002	−0.03, 0.03	0.91
Any vasopressors	291 (21)	179 (20)	112 (23)	−0.03	−0.08, 0.01	0.17
Any infusion	431 (31)	293 (33)	138 (29)	0.04	−0.01, 0.09	0.11
Having 2 infusions or more	194 (14)	124 (14)	70 (14)	−0.01	−0.05, 0.03	0.75

*Type of infusion*						
Insulin	200 (14)	136 (15)	64 (13)	0.02	−0.02, 0.06	0.32
Clevidipine	75 (5)	54 (6)	21 (4)	0.02	−0.01, 0.04	0.17
Nicardipine	71 (5)	54 (6)	17 (4)	0.03	0.002, 0.05	**0.03**
Esmolol	38 (3)	26 (3)	12 (2)	0.004	−0.01, 0.02	0.64
Any OR	763 (55)	495 (55)	268 (55)	−0.002	−0.06, 0.05	0.95
Any OR within 12 hours	328 (24)	205 (23)	123 (25)	−0.03	−0.07, 0.02	0.29
Any ECMO	23 (2)	17 (2)	6 (2)	0.007	−0.01, 0.02	0.33
Any IABP	15 (1)	10 (1)	5 (1)	0.0008	−0.01, 0.01	0.89
Any IR	29 (2)	21 (2)	8 (2)	0.007	−0.01, 0.02	0.37

*Hospital outcome, N (%)*						
Discharge home	572 (41)	358 (40)	214 (44)	−0.04	−0.10, 0.01	0.12
Acute rehab	347 (25)	239 (27)	108 (22)	0.04	−0.01, 0.09	0.07
Skilled nursing home	248 (18)	156 (17)	92 (19)	−0.02	−0.06, 0.03	0.46
Dead or hospice	214 (15)	144 (16)	70 (14)	0.02	−0.02, 0.06	0.43
Dead within 24 hours	30 (2)	19 (2)	11 (2)	−0.002	−0.02, 0.01	0.85
CCRU length of stays (hours), median (IQR)	438 [212–1051]	443 [210–1046]	448 [218–1054]	−3	−45, 38	0.87
Hospital length of stays (minutes), median (IQR)	9.1 [4.7–16.8]	9.4[5.0–17.1]	8.4 [4.4–15.6]	0.69	−0.13, 1.53	0.10
(b)						
Variables^*∗*^	OR	95% CI	*P* value	VIF		

Transfer from rural areas	0.90	0.60, 1.36	0.63	1.31		
Age	1.04	1.03, 1.06	**<0.001**	1.31		

*Past medical history*						
Hypertension	0.70	0.46, 0.99	**0.05**	1.23		

*Clinical factors at arrival: categorical*						
Mechanical ventilation	1.76	1.12, 2.77	**0.01**	1.72		

*Clinical factors at arrival: continuous (each unit)*						
SOFA	1.21	1.15, 1.28	**<0.001**	2.03		
WBC (x1000 counts per *µ*L)	1.02	1.00, 1.04	**0.02**	1.07		
Hemoglobin (g/dL)	0.92	0.86, 0.99	**0.03**	1.18		
Lactate (mg/dL)	1.13	1.05, 1.22	**0.001**	1.24		
Troponin (ng/mL)	1.01	1.00, 1.02	**0.03**	**1.05**		

CCRU, critical care resuscitation unit; CI, confidence interval; OR, operating room; ECMO, extracorporeal membrane oxygenation; IABP, intraaortic balloon pump; IR, interventional radiology; IQR, interquartile range. ^*∗*^Hosmer–Lemeshow test chi-square 12.56, D(f) = 8; *P*=0.13; AUROC = 0.85. OR, odds ratio; CI, confidence interval; VIF, variance inflation factor; SOFA, the sequential organ failure assessment; WBC, white blood cells; *µ*L, microliter; L, Liter; g, gram; mg, milligram; dL, deciliter; ng/mL, nanograms per milliliter (ng/ml); AUROC, area under the receiving operating characteristic curve. Only statistically significant variables were included. Bold values denote statistically significant comparisons.

**Table 3:** List of all variables being used in the multivariable logistic and multivariable ordinal regressions.

Continuous variables	Categorical variables
Age	Sex
Lactate	Past medical history of hypertension
Troponin	Past medical history of diabetes
White blood cell count	Past medical history of liver disease
Hemoglobin	Past medical history of kidney disease
SOFA	Past medical history of heart disease
Shock index	CCRU arrival during the weekend (Sat-Sun)
Ground distance to CCRU	CCRU arrival time of day (7am–7pm vs. 7pm–7am)
	Originating hospital type (teaching vs. nonteaching)
	Top 5 accepting services: cardiac surgery, soft tissue surgery, ACES, neurosurgery, and vascular surgery
	Transfer from a rural location
	OR within 12 hours of arrival
	Mechanical ventilation prior to arrival
	Any infusions initiated prior to arrival
	Any blood products given prior to arrival

CCRU, critical care resuscitation unit; SOFA, the sequential organ failure assessment.

**Table 4:** Multivariable logistic regression assessing the association between patients' demographic and clinical factors and hospital disposition of dead/hospice.

Variables^*∗*^	OR	95% CI	*P* value	VIF
Age	1.04	1.03, 1.06	**<0.001**	1.31
Male	0.84	0.60, 1.20	0.35	1.09

*Past medical history*				
Hypertension	0.70	0.46, 0.99	**0.05**	1.23
Liver disease	1.01	0.52, 1.95	0.98	1.12
Kidney disease	1.09	0.70, 1.70	0.71	1.15
Heart disease	0.99	0.64, 1.51	0.96	1.16
Diabetes	0.73	0.47, 1.14	0.17	1.14

*Clinical factors at arrival: categorical*				
Any infusions	1.09	0.74, 1.60	0.66	1.19
Any blood products	1.00	0.57, 1.74	0.99	1.20
Mechanical ventilation	1.76	1.12, 2.77	**0.01**	1.72

*Institutional factors*				
Rural transfer	0.90	0.60, 1.36	0.63	1.31
Arrival weekend (Sat-Sun)	1.26	0.86, 1.85	0.24	1.04
Arrival night (7pm–7am)	0.98	0.70, 1.39	0.93	1.02
Teaching facility	0.98	0.64, 1.48	0.91	1.29
OR within 12 hours	0.95	0.63, 1.43	0.81	1.05

*Clinical factors at arrival: continuous (each unit)*				
SOFA	1.21	1.15, 1.28	**<0.001**	2.03
WBC (×1000 counts per *µ*L)	1.02	1.00, 1.04	**0.02**	1.07
Hemoglobin (g/dL)	0.92	0.86, 0.99	**0.03**	1.18
Shock index	1.16	0.84, 1.60	0.38	1.08
Lactate (mg/dL)	1.13	1.05, 1.22	**0.001**	1.24
Troponin (ng/mL)	1.01	1.00, 1.02	**0.03**	1.05

^*∗*^Hosmer–Lemeshow test chi-square 12.56, D(f) = 8; *P*=0.13; AUROC = 0.85. OR, odds ratio; CI, confidence interval; VIF, variance inflation factor; SOFA, the sequential organ failure assessment; WBC, white blood cell; *µ*L, microliter; L, liter; g, gram; mg, milligram; dL, deciliter; ng/mL, nanograms per milliliter (ng/ml); AUROC, area under the receiving operating characteristic curve. Bold values are significant.

**Table 5:** Results from ordinal logistic regression assessing the association between patients' demographic and clinical factors and the likelihood of clinically significant discrepancy in the primary outcome of disposition, where 0 = home, 1 = acute rehabilitation center, 2 = skilled nursing home/facility, and 3 = hospice/death.

Variables	OR	95% CI	*P* value	Coefficient
Age	0.97	0.96–0.97	**<0.001**	−0.03
Gender: female	0.96	0.78–1.19	0.76	−0.04

*Past medical history*				
Hypertension	1.05	0.83–1.32	0.68	0.05
Diabetes	0.92	0.72–1.19	0.53	−0.08
Liver disease	1.04	0.69–1.59	0.84	0.04
Kidney disease	0.82	0.61–1.10	0.19	−0.19
Heart disease	1.00	0.77–1.31	0.97	0.004

*Clinical factors at arrival*				
SOFA	0.86	0.82–0.89	**<0.001**	−0.16
Shock index	0.88	0.68–1.12	0.29	−0.13
Troponin (ng/ml)	0.99	0.98–1.00	**0.01**	−0.01
Arrival WBC (×1000 counts per *µ*L)	0.97	0.96–0.99	**<0.001**	−0.03
Hemoglobin (g/dL)	1.03	1.00–1.07	**0.05**	0.03
Lactate (mg/dL)	0.89	0.84–0.94	**<0.001**	−0.12
Blood products initiated prior to arrival	1.43	0.98–2.08	0.07	0.35
Infusions initiated prior to arrival	0.85	0.67–1.08	0.17	−0.17
Mechanical ventilation prior to arrival	0.71	0.53–0.96	**0.03**	−0.34
Any OR in 12 hours	0.73	0.55–0.95	**0.02**	−0.31

*Accepting* *specialty*^1^				
Cardiac surgery	1.94	1.42–2.65	**<0.001**	0.66
Soft tissue surgery	1.00	0.70–1.42	0.99	−0.0003
Acute care emergency services	1.47	1.01–2.16	**0.05**	0.39
Neurosurgery	0.86	0.61–1.22	0.41	−0.15
Vascular surgery	1.67	1.11–2.52	**0.01**	0.51

*Arrival and facility demographics*				
Arrival on weekend	0.80	0.64–1.02	0.07	−0.21
Arrival at night	1.07	0.87–1.32	0.52	0.07
Teaching facility	0.73	0.56–0.95	**0.02**	−0.31
Transport from rural area	1.21	0.92–1.59	0.17	0.19
Ground distance to CCRU (km)	1.00	0.99–1.00	**<0.001**	−0.01

^1^The significance is that they are top 5 specialties. The bold values are significant values.

**Table 6:** Total number of patients and number of patients with the top 5 diagnoses being transferred to the CCRU per each rural county in the state of Maryland.

County	Total number of patients transferred to CCRU	Number of rural patients with top 5 most common diagnosis	Necrotizing fasciitis	Ischemic stroke	Aortic/arterial dissection	Pulmonary embolism	Brain bleed/intracranial hemorrhage/CVA
Anne Arundel	158	54	20	11	18	5	0
Harford	116	53	13	12	9	13	6
Talbot	70	21	4	1	4	5	7
Frederick	62	21	5	7	5	3	1
Carrol	57	11	7	0	3	1	0
Wicomico	23	13	9	1	0	2	1
Charles	18	7	0	0	2	3	2
Cecil	16	5	3	0	1	0	1
Dorchester	16	6	2	2	1	1	0
Queen Anne's	14	4	2	0	1	1	0
Allegany	11	5	0	5	0	0	0
Kent	10	2	1	1	0	0	0
Worcester	10	0	1	0	1	0	0
St. Mary's	9	1	1	0	0	0	0
Calvert	8	5	3	0	0	2	0
Somerset	2	1	1	0	0	0	0

**Table 7:** (a) Difference in mortality rates for patients transferred from rural and urban hospitals with the diagnosis of necrotizing fasciitis. (b) Difference in mortality rates for patients transferred from rural and urban hospitals with the diagnosis of ischemic stroke. (c) Difference in mortality rates for patients transferred from rural and urban hospitals with the diagnosis of aortic/arterial dissection. (d) Difference in mortality rates for patients transferred from rural and urban hospitals with the diagnosis of pulmonary embolism. (e) Difference in mortality rates for patients transferred from rural and urban hospitals with the diagnosis of intracranial hemorrhage.

(a)						
Variable	All patients	Rural	Urban	Difference	95% CI	*P* value
	*N*=178	*N*=56	*N*=122			

Dead	17 (9)	7 (12)	10 (8)	−0.04	−0.14, 0.06	0.4

(b)						
Variable	All patients	Rural	Urban	Difference	95% CI	*P* value
	*N*=113	*N*=33	*N*=80			

Dead	17 (15)	3 (9)	14 (17)	0.08	−0.04, 0.21	0.2

(c)						
Variable	All patients	Rural	Urban	Difference	95% CI	*P* value
	*N*=90	*N*=28	*N*=62			

Dead	10 (11)	1 (3)	9 (14)	0.11	−0.002, 0.22	0.05

(d)						
Variable	All patients	Rural	Urban	Difference	95% CI	*P* value
	*N*=72	*N*=33	*N*=39			

Dead	4 (5)	1 (3)	3 (8)	0.05	−0.06, 0.15	0.37

(e)						
Variable	All patients	Rural	Urban	Difference	95% CI	*P* value
	*N*=68	*N*=19	*N*=49			

Dead	15 (22)	6 (31)	9 (18)	−0.13	−0.37, 0.10	0.27

## Data Availability

The data used to support the findings of this study are not available for the public due to IRB restrictions.

## Appendix

### A. List of all Variables for Regression Analyses

List of all variables being used in the multivariable logistic and multivariable ordinal regressions in Table 3.

### B. Full Results from the Multivariable Logistic Regression

Multivariable logistic regression assessing the association between patients' demographic and clinical factors and hospital disposition of dead/hospice. All listed variables are included in the model in Table 4. Appendix generated from our own dataset for this study.

### C. Full Results from the Ordinal Regression Analysis

Results from ordinal logistic regression assessing the association between patients' demographic and clinical factors and the likelihood of clinically significant discrepancy in the primary outcome of disposition, where 0 = home, 1 = acute rehabilitation center, 2 = skilled nursing home/facility, and 3 = hospice/death in Table 5.

### D. Number of Patients from Top 5 Diagnoses and Rural Counties in the State of Maryland, United States

Total number of patients and number of patients with the top 5 diagnoses being transferred to the CCRU per each rural county in the state of Maryland in Table 6. Appendix generated from our own dataset for this study.

### E. Subgroup Analyses Comparing Mortality Rates of Patients with the Top 5 Diagnoses

Subgroup analyses on the difference in mortality rates for the top 5 diagnoses for patients transferred from rural and urban hospitals were generated from our own dataset. The top five diagnoses, listed in the order below, are necrotizing fasciitis, ischemic stroke, aortic/arterial dissection, pulmonary embolism, and intracranial hemorrhage, which are shown in Table 7. Appendix generated from our own dataset for this study.
